# Deficits in olfactory sensitivity in a mouse model of Parkinson’s disease revealed by plethysmography of odor-evoked sniffing

**DOI:** 10.1038/s41598-020-66201-8

**Published:** 2020-06-08

**Authors:** Michaela E. Johnson, Liza Bergkvist, Gabriela Mercado, Lucas Stetzik, Lindsay Meyerdirk, Emily Wolfrum, Zachary Madaj, Patrik Brundin, Daniel W. Wesson

**Affiliations:** 1 Center for Neurodegenerative Science, Van Andel Institute, Grand Rapids, MI 49503 US; 2 Bioinformatics and Biostatistics Core, Van Andel Institute, Grand Rapids, MI 49503 US; 3 Department of Pharmacology and Therapeutics, University of Florida, Gainesville, FL USA

**Keywords:** Parkinson's disease, Olfactory bulb

## Abstract

Hyposmia is evident in over 90% of Parkinson’s disease (PD) patients. A characteristic of PD is intraneuronal deposits composed in part of α-synuclein fibrils. Based on the analysis of post-mortem PD patients, Braak and colleagues suggested that early in the disease α-synuclein pathology is present in the dorsal motor nucleus of the vagus, as well as the olfactory bulb and anterior olfactory nucleus, and then later affects other interconnected brain regions. Here, we bilaterally injected α-synuclein preformed fibrils into the olfactory bulbs of wild type male and female mice. Six months after injection, the anterior olfactory nucleus and piriform cortex displayed a high α-synuclein pathology load. We evaluated olfactory perceptual function by monitoring odor-evoked sniffing behavior in a plethysmograph at one-, three- and six-months after injection. No overt impairments in the ability to engage in sniffing were evident in any group, suggesting preservation of the ability to coordinate respiration. At all-time points, females injected with fibrils exhibited reduced odor detection sensitivity, which was observed with the semi-automated plethysmography apparatus, but not a buried pellet test. In future studies, this sensitive methodology for assessing olfactory detection deficits could be used to define how α-synuclein pathology affects other aspects of olfactory perception and to clarify the neuropathological underpinnings of these deficits.

## Introduction

Hyposmia, a reduced sense of smell, affects more than 90% of all Parkinson’s disease (PD) patients, with a minority of these patients experiencing anosmia, a complete loss of their sense of smell^1,2^. This olfactory dysfunction can occur up to 10 years before the onset of motor symptoms and the clinical diagnosis of PD^3,4^. Currently, the diagnosis of PD heavily relies on the motor manifestations of the disease that correlates with substantial loss of dopamine neurons in the substantia nigra and the widespread deposition of intracellular inclusions, Lewy bodies and Lewy neurites, partly composed of misfolded α-synuclein fibrils^5^. These protein aggregates are able to seed the aggregation of endogenous protein and propagate to interconnected areas (reviewed in^6^). According to the Braak staging hypothesis, based on the analysis of post-mortem tissue from PD patients, α-synuclein pathology is initially present in the dorsal motor nucleus of the vagus, as well as the olfactory bulb (OB) and the anterior olfactory nucleus (AON). Later it is presents widely throughout the forebrain and eventually it affects the cerebral cortex^7–9^. The presence of α-synuclein pathology in olfactory structures could explain the olfactory dysfunction seen in PD patients^10–12^.

Animal models of PD-relevant olfactory dysfunction have been established using neurotoxins including, 1-Methyl-4-phenyl-1,2,3, 6-tetrahydropyridine (MPTP) and 6-hydroxydopamine (6-OHDA) (for an extensive review, see^13^). While these models produce neurodegeneration and induce olfactory dysfunction, neither yield robust intraneuronal deposition of misfolded α-synuclein. Genetic models of PD may be used to study olfactory dysfunction, however in these models the olfactory deficits can take a long time to develop^13^, and genetic forms of the disease only account for a small percentage of PD cases (less than 10%)^14^. The mechanism of α-synuclein propagation has been used to reproduce some of the most distinctive hallmarks of PD in mice by the injection of recombinant α-synuclein preformed fibrils (PFFs) (reviewed in^15^). The injection of PFFs into the OB of wild type mice causes Lewy Body-like pathology, which propagates to synaptically connected brain regions over several months^16–19^, including key olfactory areas implicated in odor perception. Additionally, these mice have been reported to develop progressive olfactory deficits for odor retention/memory and detection thresholds, using a testing set-up involving a cartridge containing a paper swab impregnated with odor^16^. In a more recent report, PFFs injections in the OB and/or AON of wild type mice were reported to trigger Lewy Body-like pathology accompanied by sex and age-dependent behavioral deficits, including olfactory dysfunction assessed with the buried pellet test^20^.

Until now, olfactory deficits have been evaluated in PD animal models using several approaches, such as the buried pellet test, social-odor discrimination test, and odor habituation/dishabituation test^16,20–23^. While these tests have the advantage of being simple with no requirements for a special apparatus or behavioral shaping, several aspects of them present possible downsides. These can conceptually be synthesized into two main issues: 1) stimulus control, and 2) behavioral monitoring. First, regarding stimulus control, in the assays described above, liquid odor is placed on a substrate (i.e., filter paper, cotton swab), or a food pellet is presented to the animal. While the liquid dispensed onto the substrate is of known intensity, the vapor released is not; thus uncertainty is left in terms of stimulus intensity. Odorant composition of food stimuli is also unstable and may differ considerably from item to item. Second, to monitor behavior in these assays, the observer often manually times and interprets the animal’s response to the odor or food pellet, e.g., when the animal’s snout is within 1 cm of the object containing the odor. While video is often recorded for later scoring, the fact that the animal’s snout is not within a given distance from the odor does not necessarily mean the animal is not investigating the plume (via sniffing). Due to the variability observed using some of the olfactory tests mentioned above, a large number of animals is usually required to detect differences between groups. Taken together, these methods for assaying odor detection and investigation could lead to difficulties in reproducing results and also make it difficult to sensitively define olfactory deficits.

In the present study, we show that bilateral OB injections of PFFs cause olfactory detection deficits in female wild type mice one-, three- and six-months post-PFFs injection using a sample size of only 5-6 animals per group. To achieve this, we monitored respiration for changes (or lack thereof) in odor-evoked sniffing which is reflexively displayed by rodents upon detection of a novel stimulus. To monitor sniffing, we adapted a plethysmograph and olfactometer based upon the methods of Wesson *et al*.^24^, Hegoburu *et al*.^25^, and Youngentob^26^. Using this apparatus, animals were delivered progressively increasing intensities of odor vapors in an ascending staircase design. These results, which sensitively and rigorously define odor detection sensitivity deficits following OB injections of PFFs, add to our understanding of the pathological mechanisms that might underlie olfactory deficits in PD.

## Methods and materials

### Animals

Ten to twelve-week-old male and female C57BL/6N mice were sourced from the Van Andel Institute vivarium. These mice were housed with a maximum of four mice per cage under 12-h light/12-h dark cycles with free access to food and water. The housing of animals and all procedures were performed in accordance with the *Guide for the Care and Use of Laboratory Animals* (United States National Institutes of Health) and were approved by the Van Andel Institute’s Animal Care and Use Committee.

### PD model by bilateral injections of PFFs into the olfactory bulbs

Mouse α-synuclein amyloid aggregates were produced as described in^27^ and were kindly provided by Dr. Kelvin Luk, University of Pennsylvania Perelman School of Medicine, USA. Before surgery, PFFs were produced by the sonication of α-synuclein amyloid aggregates in a water-bath cup-horn sonicator for four min (QSonica, Q700 sonicator, 50% power, 120 pulses at 1 s ON, 1 s OFF) and were maintained at room temperature until injection. Mice were anesthetized with isoflurane and injected bilaterally in the OB with either 0.8 µL of PFFs (5 µg/µl; *n* = 6 females, *n* = 5 males) or 0.8 µL of PBS (phosphate buffered saline) as a control (*n* = 5 females, *n* = 6 males) (coordinates from bregma: AP: +5.4 mm; ML: +/−0.75 mm and DV: −1.0 mm from dura). Injections were made at a rate of 0.2 µL/min using a glass capillary attached to a 10 µL Hamilton syringe. After injection, the capillary was left in place for three min before being slowly removed. Prior to incision, the animals were injected with local anesthetic into the site of the future wound margin (0.03 mL of 0.5% Ropivacaine; Henry Schein, USA). Following surgery mice received 0.5 mL saline s.c. for hydration, and 0.04 mL Buprenex (Henry Schein, USA) s.c. for analgesia.

### Semi-automated system for assaying mouse odor perception

**Figure 1:**
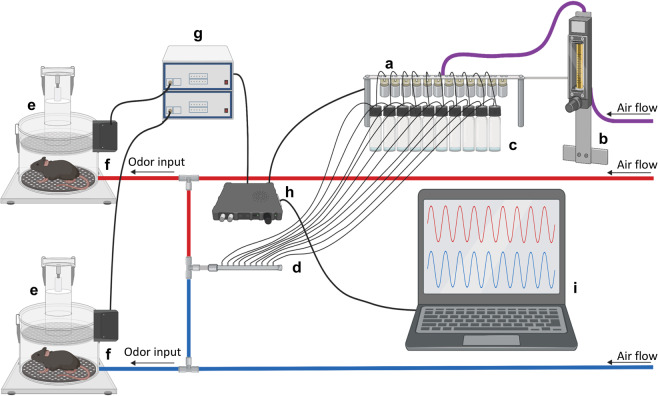
Semi-automated system for assaying mouse odor perception. The olfactometer contains a series of valves (**a**) that can be digitally triggered through software to open, allowing a set air flow, regulated by a flow meter (**b**), to pass through the associated valve into the connected odor vial (**c**). This airflow will continue for 6 s, during which the air flow moves from the headspace of the vial containing liquid odor through the tubing to combine with the constant air flow in a manifold (**d)** and finally on towards the animal in each plethysmograph chamber (**e**). The odor enters beneath the perforated floor of the plethysmograph chamber and the chamber’s exhaust vent is towards the chamber ceiling. The changes in air pressure are recorded through the connected flow transducer (**f**). This signal is amplified and filtered (**g**) before being digitized (**h**) and stored to the computer. Respiration and valve timing are both recorded digitally allowing monitoring of the animal’s respiration in real-time (**i**). Image created with BioRender.

A schematic of the semi-automated olfactory testing set-up, adapted from^26^ can be found in Fig. 1. Product details for the equipment required to create this apparatus are available in Supplementary Table 1. The concept of the apparatus is to enable monitoring of odor-evoked sniffing mice display upon detection of a novel odor. To accomplish this, two main components need to be in place. First, there needs to be a method for monitoring respiration/sniffing behavior of the mice. To measure sniffing, we used unrestrained whole-body plethysmography to monitor respiration of the mice as they freely explored the plethysmograph chamber (Data Sciences International). Respiratory transients were detected using a Data Sciences flow transducer and digitized (0.1–20 Hz) at 600 Hz in Synapse Lite (Tucker Davis Technologies) following a 100X gain amplification (Cygnus Technology Inc). Positive pressure of room air was applied to the chamber using a stable-output air pump (Tetra Whisper).

The second main component of the apparatus is an olfactometer allowing precise control of odor delivery. For this, we constructed an odor presentation device (*viz*., an ‘olfactometer’) adapted from the design of Gadziola *et al*.^28^. Briefly, custom code written in Synapse allowed a user to open one of many relay valves, each when opened allows the flow of clean air into a glass odor headspace vial filled with 1 ml of odor (delivered for 6 s duration; odors and liquid dilutions defined below). This then results in a stream of odor vaporized air (50 mL/min, mixed with 1 L/min from the pump) through chemically-resistant Teflon tubing into the plethysmograph chamber. The output from the odor vial was split with a 3-way connector thereby affording the ability to deliver vaporized odor to two plethysmograph chambers simultaneously (Fig. 1). The odor was delivered in-line with the breathable room air, into the bottom of the plethysmograph. This resulted in odor coming through the plethysmograph’s perforated floor up towards the animal as it proceeded to the exhaust outlet positioned near the chamber ceiling. Upon user initiation of an odor trial, numerous auxiliary valves not gating odor flow would change states which provided acoustic stimuli and possibly other cues to prohibit the mice from associating specific multi-sensory cues with a possible odor and/or becoming aroused by one specific multi-sensory cue and thus influencing their respiration (which is strongly influenced by arousal). Using this set-up, in a longitudinal design, mice underwent olfactory detection sensitivity testing one-, three- and six-months post-PFFs or PBS injection.

### Habituation prior to testing

Animals were habituated to the testing apparatus for three days prior to testing for each timepoint (months post-injection) so they could acclimate to the plethysmograph chamber, noises, and possible mechanosensory/pressure changes which may be associated with valve opening and closing. For habituation the animals were placed in the plethysmograph for 30 min with mineral oil vapor (odor vehicle, purchased from Sigma Aldrich) presented once per min (6 s each).

### Odor detection testing

Following the habituation described above, animals were presented with mineral oil once per min for 11 min, then exposed to increasing concentrations of heptanal, starting with 10^−8^ and proceeding in intensity to 10^−2^, also triggered once per min. This protocol was repeated with methyl valerate, isoamyl acetate, and 1,7-octadiene on subsequent days. All odors were acquired at their highest available purity from TCI Co. Ltd, USA and were diluted with mineral oil (Sigma Aldrich) to 2 Torr vapor pressure before their serial liquid dilution.

**Figure 2:**
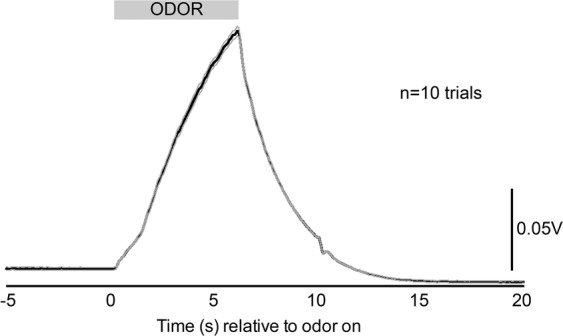
Dynamics of odor delivery into the plethysmograph. Photoionization detector acquired average traces of 10 trials of the odor 2-heptanone (mean ± SEM). Timing of odor delivery indicated by gray shaded horizontal bar. The detector sampled air from within the plethysmograph thereby assessing the onset and evacuation of the odor upon and following odor delivery, respectively.

To validate the latency of odor entry into the chamber as well as evacuation of odor from the chamber following trials, we used a photoionization detector to monitor volatile intensity (Aurora Scientific, miniPID). For these recordings, the detector was placed to sample air from within the plethysmograph’s base which would approximate where the animal’s snout would mostly be during testing. Figure 2 shows the odor dynamics of 2-hepatone averaged over 10 repeated trials (duration and inter-stimulus interval as described above). In this example, it is evident that odor begins to arrive within the chamber a few hundred ms following onset (valve opening) and rises gradually in intensity (parts per million, indicated by the detector in V) as the odor replaces clean air for space within the chamber. After odor offset there is a sharp decline leaving no detectable odor within ~10 s (Fig. 2). This is consistent with the exchange rate of clean air entering the plethysmograph to that of odor introduced. These data support the use of a 1 min inter-stimulus interval which would ensure that between each odor trial the air in the chamber is void of detectable odor.

### Analysis of respiratory data

Respiratory data were analyzed in Spike2 software (Cambridge Electronic Design Ltd) using a custom script to quantify the percent of time spent in investigatory sniffing after odor-onset. First, common noise rejection was applied to the digital signal from each chamber’s transducer. Following, each signal was digitally filtered (1 Hz to 15 Hz Butterworth, 2^nd^ order), and inspiration peaks were detected by means of identifying the maximum point of each cycle. Instantaneous respiration frequency was calculated off of these peaks and these data were then down-sampled to 100 Hz for ease of handling. The time mice spend sniffing above 6 Hz is considered investigatory behavior^29^, as the minimum range of respiration frequency associated with odor investigation in mice is, generally, 6-7 Hz^30^. Therefore, off of the instantaneous frequency data, we calculated the duration of time (out of 8 s of total time following odor delivery) that the animals’ instantaneous respiration frequency exceeded 6 Hz as a measurement of odor investigation. While the odor valve was only open for 6 s, we analyzed an 8 s period which allowed animals sufficient time to possibly display sniffing reflexively depending upon whether or not they detected an odor. This 8 s period began 1 s after the odor valve opened to allow time for the odor to reach the testing chamber (Fig. 2). This 1 s lag in the start of the analysis period also was selected to exclude the brief (<500 ms) pressure artifacts which may occur upon the onset of odor delivery from influencing accurate detection of respiratory events. In a subset of analyses, we also quantified the instantaneous respiratory frequencies from each animal during a pre-odor period (−20 s to 0) and used these to identify the most common mode of sniffing per mouse.

### Zinc sulfate hyposmia model

As previously described^31,32^, hyposmia was induced by bilateral intranasal injections with 20 μL of 5% (0.17 M) zinc sulfate (Sigma Aldrich) in male C57BL/6N mice. To achieve this, a 25 μL Hamilton syringe (Hamilton) with a 26 G needle (Hamilton) was fitted with the narrow part of a microloader tip (Eppendorf). The latter provided a long flexible ending allowing easy insertion 4 mm into the naris of the mouse so that the solution could saturate the nasal turbinates. Mice were anesthetized with i.p. ketamine (120 mg/kg; Henry Schein, USA)/xylazine (16 mg/kg; Santa Cruz Animal Health, USA). Once unresponsive, 10 µL of solution was slowly injected over 1 min into the first naris, and the mouse was then immediately inverted (nose pointing down) for 1.5 min to minimize the mouse ingesting the solution, which can cause systemic effects. The mouse was next laid on the injected side for 2 min then on its back for 2 min before the process was repeated with the other naris (Fig. 4a). After both nares had been injected twice (total of 40 µL solution) mice were administered i.p. Antisedan (Santa Cruz Animal Health, USA) 1 mg/kg, to reverse the effects of xylazine. Control mice underwent the same procedure except they received 20 µL of 0.9% saline per naris in place of the zinc sulfate. All mice were allowed 48 h to recover prior to odor detection sensitivity testing in the plethysmograph as described above. Of the *n* = 14 mice that received zinc sulfate and *n* = 10 mice that received saline, *n* = 5 mice (35.7% mortality rate) and *n* = 1 mouse (10% mortality rate) required premature euthanasia due to welfare reasons, respectively. This resulted in both groups having *n* = 9 mice for the post-injection olfactory analysis.

### Statistics on respiratory data

Linear mixed-effects models with square-root transformations were run in R version 3.6.0 to assess overall average instantaneous frequency at each timepoint within each sex. Square-root transformations improved model diagnostics for both the normality of residuals and homoscedasticity. For this analysis random effects for mouse and dilution were used, and odor was included as a covariate. For comparing treatment differences within each odor dilution and sex at each timepoint post-injection (age) we used a linear mixed-effects model that tested instantaneous frequency by treatment adjusted for sex and odor. For both analyses, linear contrasts were used test the specific hypotheses of interest and p-values were corrected for multiple testing using the Benjamini-Hochberg method.

### Buried pellet test

The buried pellet test was performed for three consecutive days, with 12 h of overnight fasting in between. Each day of testing, the location of the buried food was changed. The testing paradigm was adapted from Lehmkuhl *et al*.^21^. The buried pellet testing was performed one week prior to the semi-automated olfactory testing described above.

Three days prior to the first day of testing, food was restricted for 12 h during the active phase for mice (dark cycle). When the mice had access to food, they were also given one piece of sweetened cereal (Cap’n Crunch) to avoid neophobic food behavior during testing. The starting weight of the animals was recorded prior to food restriction; if an animal lost 10% of its starting weight it was exempt from food restriction and subsequent testing.

For testing, animals were moved to individual clean cages containing 2 cm of fresh bedding, covered with a filter top lid, and allowed to acclimate for 5 min. After acclimation, the mouse was put in a clean holding cage while the researcher buried a sweetened cereal item 0.5 cm under the bedding in the testing cage. The mouse was then placed back in the test cage and the latency for the mouse to uncover and put its nose against the food was measured. Once the food was uncovered, the mouse was allowed to eat it. The maximum time allowed to find the food was 5 min; if a mouse was unable to find it within that time period, its latency was recorded as 300 s and it was given the food. Tested mice were not allowed back into their housing cage if it contained untested mice. When all animals in a cage had been tested, they were given free access to food. To avoid outside olfactory cues, the experimenter used double pairs of gloves and changed the outer pair in between mice.

To control for variability in motivation for food seeking behavior, the surface pellet test was also carried out. This was performed as described for the buried pellet test, but instead the food item was placed on the surface of the bedding. If a mouse was uninterested in the food item, it was excluded from subsequent testing. All mice in our study were motivated by this sweetened cereal.

### Statistics on buried pellet test data

The latency for the mice to uncover the food item was pooled from the three trials and an unpaired Student’s T test was performed in GraphPad Prism version 6.

### pSer129 α-synuclein pathology scoring

Six-months post-PFFs injections animals were anesthetized with sodium pentobarbital (130 mg/kg; Sigma Aldrich) and perfused through the ascending aorta with isotonic saline followed by ice-cold 4% paraformaldehyde (pH 7.4). Brains were removed, post-fixed overnight at 4 °C in 4% paraformaldehyde and subsequently placed in 30% sucrose and stored at 4 °C until sectioning. Brains were frozen and coronal sections of 40 µm containing the AON, piriform cortex (PCx) and substantia nigra were cut on a sliding microtome (Leica, Germany) and collected as serial tissue sections spaced by 240 µm.

For immunohistochemistry, a series of coronal free-floating tissue sections were stained by immunohistochemistry using a primary antibody directed against pSer129 α-synuclein at 1:10,000 (Abcam, Ab51253) and goat anti-rabbit biotinylated secondary sera at 1:500 (Vector Laboratories, BA-1000). For the detection of the antibody with DAB, we used a standard peroxidase-based method (Vectastain ABC kit and DAB kit; Vector Laboratories). After dehydration, slides were coverslipped with Cytoseal 60 mounting medium (Thermo Fisher Scientific).

Slides were scanned using an Aperio AT2 scanner (Leica). As previously described^16^, the serial sections including the entire AON, PCx and substantia nigra spaced by 240 µm were scored, in a blinded manner, based on the density of α-synuclein pathology assessed as the load of pSer129 α-synuclein immunopositive signal. Briefly, each section of the region of interest was allocated a value of 0 (no pathology) to 5 (very dense pathology), in increments of 0.5, depending on the pathology load present. The mean score value for each brain region was then calculated for each animal. Non-parametric permutation tests were used to compare the group means of PBS and PFFs injected mice for each region of interest. P-values were adjusted for multiple testing via the Benjamini-Hochberg false discovery rate correction.

## Results

### pSer129 α-synuclein accumulates in the AON and the PCx

**Figure 3:**
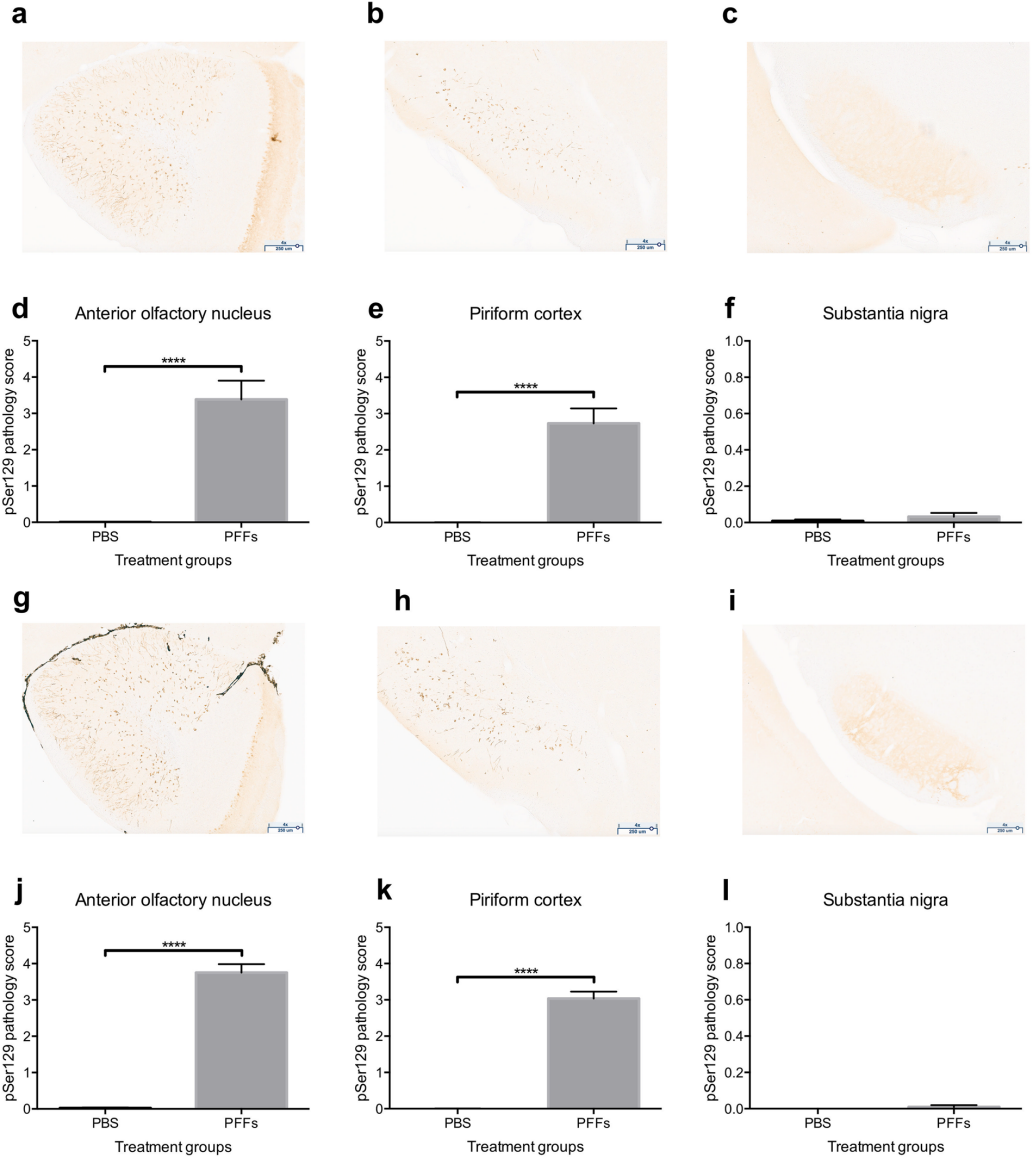
pSer129 α-synuclein pathology in mice injected with PFFs. Wild type mice were injected bilaterally in the OB with PFFs or PBS (control). Six-months after injections the load of pathological phosphorylated α-synuclein (pSer129) was assessed by immunohistochemistry. Representative images of the anterior olfactory nucleus, piriform cortex and substantia nigra of a PFFs injected female mouse (**a**, **b** & **c**) and male mouse (**g**, **h** & **i**) are shown at 4X magnification (scale bar 250 μm). The pSer129 immunopositive signal was scored on blinded serial sections including the entire anterior olfactory nucleus, piriform cortex and substantia nigra in female (**d**, **e** & **f**) and male (**j**, **k** & **l**) mice. Data displayed as mean ± SEM, n = 5-6 per treatment group. ****p ≤ 0.0001.

Immunohistological assessment six-months post-PFFs injection showed a robust accumulation of phosphorylated, pathological α-synuclein (pSer129) in key olfactory areas, including the AON (females *p* = 3e-07, Fig. 3a,d; males *p* = 1e-07, Fig. 3g,j) and the PCx (females *p* = 3e-07, Fig. 3b,e; males *p* = 1e-07; Fig. 3h,k**)**. These structures both receive mono- and bi-synaptic input from the OB^33–35^. Previous literature^16,17^, reported no accumulation of pSer129 α-synuclein in the substantia nigra of mice at this timepoint after the injection of PFFs in the OB. We sought here to also assess an absence of pSer129 α-synuclein in the substantia nigra since pathology in this structure may influence respiratory motor control. Female (Fig. 3c,f) and male mice (Fig. 3i,l) displayed no/negligible accumulation of pSer129 α-synuclein in the substantia nigra. Similarly, PBS injected control mice had no/negligible amounts of pSer129 α-synuclein pathology in all brain regions analyzed.

### Validation of the semi-automated olfactory test set-up using an established hyposmia model

Rodents display active investigation of novel odors upon their perception, which can be quantified by increases of respiratory rate (>6 Hz) into the stereotyped high frequency respiratory behavior we commonly refer to as ‘sniffing’^30,36–38^. We reasoned that impaired odor detection sensitivity would be quantifiable by reduced time during odor presentations that mice spend engaged in sniffing when exposed to increasing odor concentrations. In other words, mice will not display and/or will display less active sniffing for an odor they have yet to detect, but upon detecting an odor for the first time (as in the case for when receiving an odor of sufficiently detectable intensity), the mice will display sniffing.

**Figure 4:**
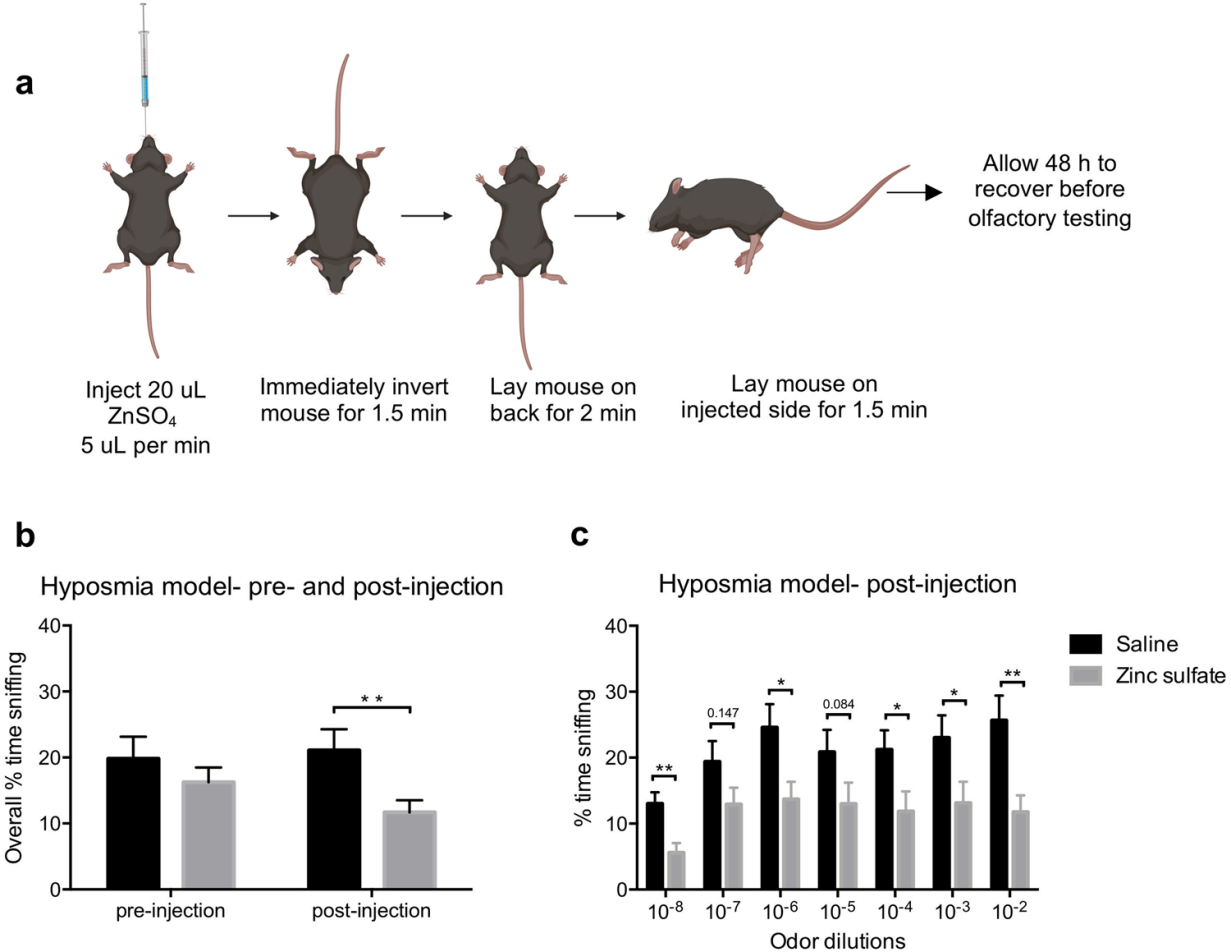
Validation of the semi-automated olfactory test set-up. Schematic describing how intranasal ZnSO_4_ injections were performed (**a**). Graphs b & c represent pooled results in response to heptanal, isoamyl acetate, methyl valerate and 1,7-octadiene odors. Male wild type mice receiving zinc sulfate spent significantly less time engaged in investigatory sniffing (**b**), as well as less time for 10^−8^, 10^−6^, 10^−4,^ 10^−3^ and 10^−2^ odor dilutions compared to the saline group (**c**). Data displayed as mean ± SEM, n = 9 per treatment group. *p ≤ 0.05, **p ≤ 0. 01. Image created with BioRender.

To validate that this semi-automated olfactory testing set-up detects reductions in olfactory sensitivity, we used an established method to generate mice that are hyposmic. Thus, we gave wild type mice intranasal zinc sulfate injections to induce temporary hyposmia^31,32^, or saline injections as control treatment (Fig. 4a). Prior to intranasal injection, there was no significant difference between mice allocated to the saline or the zinc sulfate group (Supplementary Fig. 1). After intranasal injections, mice exposed to zinc sulfate spent significantly less time engaged in overall investigatory sniffing (9.4% mean difference, *p* = *0.003*), indicating hyposmia had been induced (Fig. 4b). More specifically, zinc sulfate-injected mice spent less time engaged in investigatory sniffing for the 10^−8^, 10^−6^, 10^−4,^ 10^−3^ and 10^−2^ odor dilutions when compared to the saline group (Fig. 4c). After verifying the sensitivity of this plethysmograph-based testing set-up to detect hyposmia, we next used our apparatus to assess odor detection sensitivity in mice injected with PBS or PFFs.

### Injection of PFFs leads to olfactory detection deficits without affecting respiratory behavior

After validating the plethysmograph-based testing set-up, we explored possible progressive impairments in odor detection sensitivity in wild type female and male mice following bilateral PFFs or PBS injections in the OB. Mice were evaluated in the plethysmograph as above to assess the animal’s odor detection behavior one-, three-, and six-months post injection. As a comparison to more traditional methods, all mice were also tested in the buried food pellet test^21^. Animals from each treatment group were tested in a pseudo-random manner.

**Figure 5:**
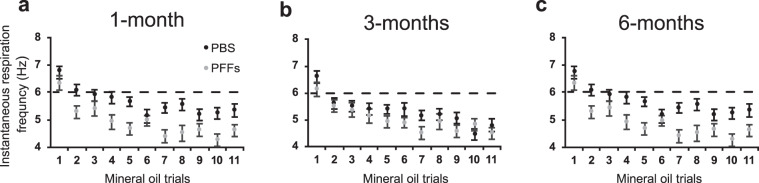
All experimental groups of mice are capable of displaying investigatory sniffing upon novel stimulus presentation which then habituates over repeated trials. The instantaneous respiration frequency during the mineral oil presentations was calculated by the same method used to derive the time spent engaged in investigatory sniffing during odor presentations. Graphs A-C show that female mice in both treatment groups (PBS - black and PFFs - gray) respond with investigatory sniffing upon the first mineral oil trial which then habituates over the repeated exposures at one- (**a**), three- (**b**) and six-months (**c**) post- injection. Horizontal dashed line = 6 Hz respiratory frequency indicator.

Before assaying odor detection abilities, we first sought to confirm through several analyses the threshold whereby to identify ‘investigatory sniffing’ in the mice and also, importantly, to establish that injections of PFFs in the OB and the seeding of the endogenous protein does not impair the basic ability of mice to respire and to display investigatory sniffing. During the 11 exposures of mineral oil in the course of the first phase of the testing paradigm (which was designed to yield habituation), all female mice regardless of treatment group, responded to the initial mineral oil exposure with investigatory sniffing (>6 Hz) at all three time points (Fig. 5a, b, c, comparing Hz during trial #1). This indicates that the mice, including PFFs-treated mice, are capable of the motor act of sniffing and as expected, they display investigatory sniffing upon detection of novel/arousing stimuli. Following, respiratory frequency decreased over repeated presentations of mineral oil to fall below 6 Hz (Fig. 5a, b, c) suggesting habituation to the presentation of mineral oil across all groups. From this point onwards in a habituated animal, we reason that increases above 6 Hz when a valve is triggered would most likely be due to the animal responding to detection of an odor, versus for instance extraneous cues associated with valve opening. As additional proof-of-concept that the basic abilities of mice to respire and sniff are not perturbed, we identified the most commonly displayed respiratory frequency from each group of animals during the baseline period before each of the odor concentrations presented. Prior to odor onset, mice injected with PFFs did not significantly differ from those injected with PBS in terms of their most commonly displayed respiratory frequency (*p* > *0.05*, within time points group comparisons, data not shown).

**Figure 6:**
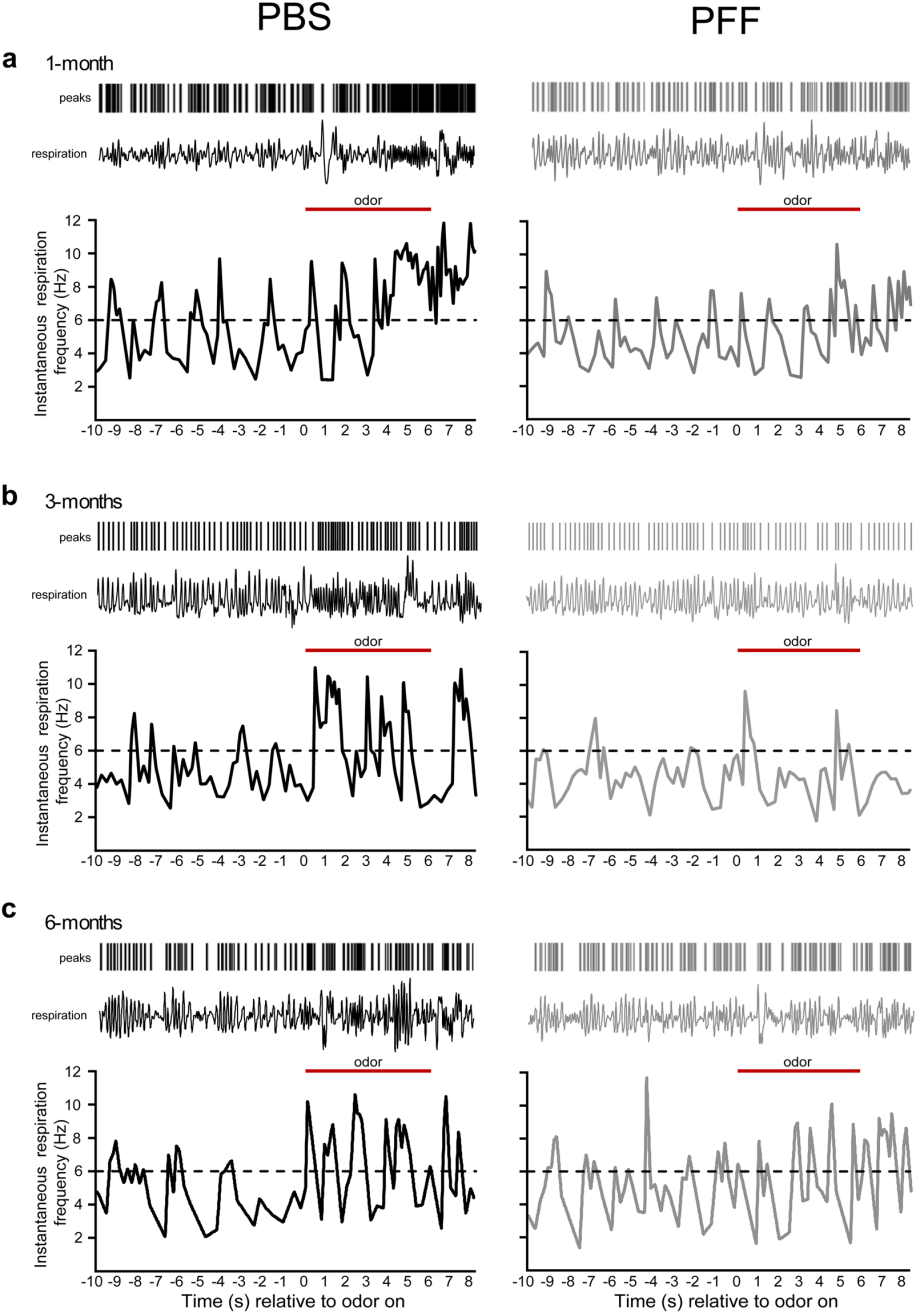
Sniffing behavior of mice injected with PBS or PFFs. Example respiratory behavior relative to odor presentation for female mice injected with PBS (black traces) or PFFs (gray traces) at one- (**a**), three- (**b**) and six-months post-injection (**c**). Rasters in each panel (top) represents inspiration peaks detected offline of digitally acquired respiration signals from the plethysmograph (1 Hz to 15 Hz Butterworth, 2^nd^ order). Plotted in the lower aspect of each panel is the instantaneous respiration frequency which was calculated off of the inspiration peaks to enable analysis of respiratory frequency dynamics relative to odor onset. Horizontal dashed line indicates the threshold operationally defined as ‘investigation behavior’ (6 Hz). Odor presentation (6 s) is indicated by the solid horizontal red bar. Examples traces are taken across odors from 1×10^−3 or −4^ dilutions (1-month = heptanal, 3-months = 1,7-octadiene and 6-months = isoamyl acetate).

Next we moved on to explore possible progressive impairments in odor detection sensitivity in these same mice. Odor perception is well known to elicit vigorous sniffing behavior in mice^30,36–38^. Figure 6 shows examples of respiration and/or sniffing of mice across all 6 experimental groups (Fig. 6a, b, c). Prior to odor-delivery the mice only occasionally displayed investigatory sniffing (>6 Hz, likely due to spontaneous non-odor driven investigation in the chamber). In these examples, odor-delivery often evoked noticeable increases in sniffing frequency for moments during the odor period. Further, at least in the PBS-injected mice, the displays of investigatory sniffing tended to last for considerable amounts of time during the odor delivery period.

**Figure 7:**
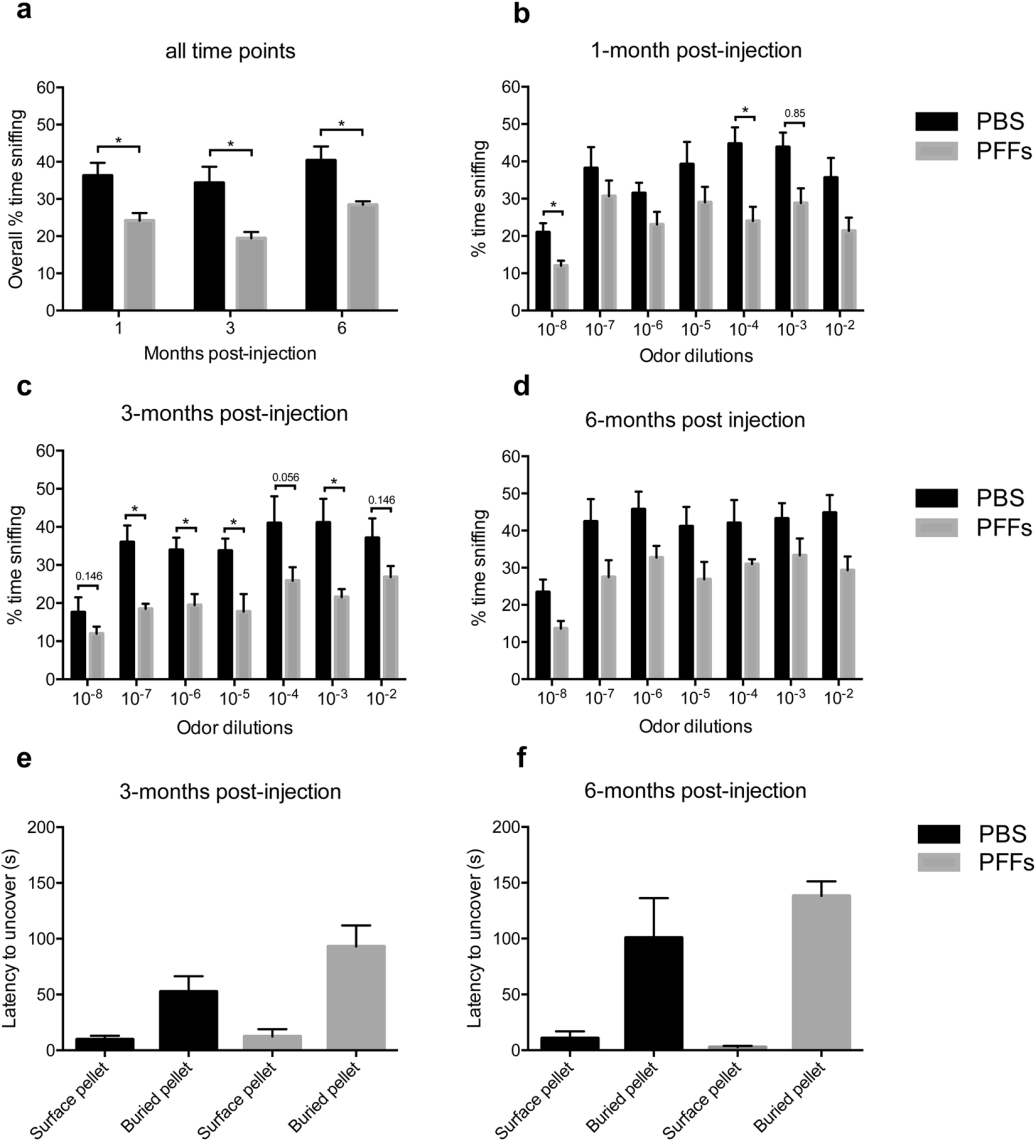
Odor detection deficits in female mice injected with PFFs. Female wild type mice receiving bilateral injections of PFFs into the OB spent significantly less time engaged in investigatory sniffing (**a**) compared to the PBS group at one- (**b**), three- (**c**) and six-months (**d**) post-injection. Graphs a-d represent pooled results in response to heptanal, isoamyl acetate and 1,7-octadiene odors. No difference was detected for mice receiving bilateral OB injections of PFFs using the buried pellet test at three- (**e**) and six-months (**f**) post-injection. Data displayed as mean ± SEM, n = 6 for the PFFs group and n = 5 for the PBS group. *p ≤ 0.05.

While capable of displaying investigatory sniffing (*e.g*., for trial # 1 of the mineral oil data presented above), female mice injected with PFFs spent significantly less time engaged in investigatory sniffing (Fig. 7a) when compared to the PBS group at one- (12.1% mean difference, *p* = *0.024*, Fig. 7b), three- (14.9% mean difference, *p* = *0.018*, Fig. 7c), and six-months post-injection (11.9% mean difference, *p* = *0.018*, Fig. 7d). This deficit was most prominent at the three-month time point where mice injected with PFFs spent significantly less time engaged in investigatory sniffing for the majority of the odor dilutions (10^−7^, 10^−6^, 10^−5,^ and 10^−3^; Fig. 7c). No significant difference was detected between the female PBS and PFFs group at three- and six-months post-injection using the buried pellet test (Fig. 7e, f). At no time point was there a significant difference between male mice injected with PFFs or PBS, when evaluated with either the semi-automated olfactory testing set-up (Supplementary Fig. 2a, b, c & d) or the buried pellet test (Supplementary Fig. 2e & f).

## Discussion

We sought to define the influence of bilateral injections of PFFs into the OB, and the subsequent pathological α-synuclein accumulation in the olfactory system, on the ability of mice to detect odors. Our results provide new insights into the influence of spreading of α-synuclein pathology on specific abilities of mice to respond to odors at a range of intensities in a behavioral paradigm developed to provide quasi-psychophysical assessments of odor detection sensitivities – a feature reported to be impacted in PD clinical populations^39^.

### pSer129 pathology in the olfactory system following bilateral PFFs injections in the OB

Six months after bilateral injections of PFFs into the OB of female and male mice we observed extensive accumulation of phosphorylated α-synuclein in the AON and PCx. These brain regions are secondary olfactory structures, which receive monosynaptic input from mitral cells and tufted cells located in the mitral cell layer and external plexiform layer of the OB, respectively^34,35^. The spread of PFFs from the OB to these and other connected brain structures has previously been well characterized following unilateral OB injections^16,17^. While pathology was not assessed at 1- and 3-months post-injection in the current study, one can assume that the pathological load would be similar for both hemispheres to that reported previously for ipsilateral brain regions following unilateral injections^16,17^. Rey *et al*.^16^ demonstrated already at 1-month post-PFFs injection there is moderate accumulation of phosphorylated α-synuclein within the AON and PCx. The amount of pathology continues to increase overtime in these two brain regions of interest, with the pathology load peaking in the AON between 6-12 months and in the PCx 12-18 months after injection of PFFs^16,17^.

As expected for our PFFs OB injection model of prodromal PD, neither female nor male mice had accumulation of phosphorylated α-synuclein in the substantia nigra at 6-months post-PFFs injection. Lack of pathology in this brain region combined with no obvious respiratory-motor impairments in these mice, including both spontaneous respiratory behavior comparable to PBS-treated mice, and preserved abilities to display investigatory sniffing to a novel stimulus (*e.g*., mineral oil trial #1, Fig. 5), supports our interpretation that the deficit in investigatory sniffing in PFFs-treated female mice is likely due to an olfactory sensory deficit rather than a deficit in the motor act of sniffing itself. This confirmation is important since respiratory-motor deficits are implicated in the hyposmia observed in PD patients^40^ and also has been recapitulated following 6-OHDA lesions of the striatum in rat models of PD^41^.

### Insights into olfactory perception in the context of synucleinopathy

In PD, olfactory dysfunction is an early non-motor symptom. While there are several non-motor signs and symptoms of PD, including mood, gastric, and sleep disorders, olfactory deficits may be more readily tested for, at a low cost, when compared to other non-motor symptoms offering a potential early diagnosis value for PD^42^. Olfactory dysfunction has been observed in several rodent models of PD^16,17,20,43^. However, in the field of neurodegenerative research, olfactory dysfunction in rodents is usually evaluated using systems which are not semi-automated. These methods may have allowed inter-personal differences between observers, such as the interpretation of an animal’s behavior and the observers’ reflexes to manually time specific events. We show that our semi-automated plethysmograph-based set-up can detect deficits in odor detection in an α-synuclein-based model of prodromal PD, while the buried pellet test, a widely used manual test, did not detect any differences between these same groups.

While this is not the first study to use an olfactometer and plethysmograph chamber-based system to assess olfaction in rodents, it is the first to apply this type of semi-automated analysis in the context of measuring olfactory impairments in a model of PD. The semi-automated system herein appears more sensitive, and therefore capable of detecting smaller changes in olfactory perception that may go undetected using manual testing. While not performed in the present study, this set-up is amenable for assaying other aspects of olfaction, including odor discrimination and generalization, for instance by employing an odor cross-habituation testing paradigm^44,45^. There are several notable aspects of behavior we uncovered herein worthy of specific discussion.

First, our findings that female mice injected with PFFs display reduced odor-evoked sniffing upon detection of a novel odor (despite intact motor abilities to display investigatory sniffing) supports the evidence that α-synuclein pathology in olfactory structures may cause olfactory deficits in mice^16^. We interpret this phenotype in the ascending staircase behavioral task as a reduction in odor detection. Notably, this impairment was detectable with our apparatus when using only 5-6 female mice per group, which is less than half of the number used in previous studies using manual-based tests^16^.

Second, male PFFs seeded mice did not display the same olfactory deficit as observed in the females. Considering that both male and female mice had a similar degree and location of α-synuclein pathology at 6-months post-PFFs injection in the current study, it raises the question of what other factors may be contributing to the olfactory deficits observed in female mice. Our results contrast Mason *et al*.^20^, in a different task, who observed fibril treated males had greater olfactory deficits than females. Sex hormones influence odor perception in rodents^46,47^ and further investigation into how sex and/or gonadal hormones influence perception in this model is necessary to understand the different olfactory outcomes reported following injection of PFFs in different studies. Two recent meta-analyses in humans report that females generally outperform males during olfaction testing, however one reported effect sizes were notably small^48^ and the other reported the effect was evident in young adults (18-50 years) but not in juvenile or aged cohorts^49^. For all of the time points of olfactory testing in this study mice were in mature adulthood or later, with the final time point (6-months post-PFFs) mice were 9-months of age, which is considered between mature adulthood and middle-aged rather than aged for a mouse. Therefore, the mice human age equivalent falls within the young adult period when females clinically display olfactory superiority. This may explain why we see that PFFs treatment in female mice is sufficient to impair olfactory detection compared to female controls and compared to males. Finally, related to the issue of sex differences in olfactory perception, we only performed ZnSO_4_ lesions in male mice to validate that the changes in ‘odor-evoked’ sniffing were truly due to odor. Future work to explore how both sexes of mice may differentially display sniffing following ZnSO_4_ lesions would be helpful in better informing how sex contributes to odor investigation, especially in hyposmic states.

Third, the data in Fig. 7 show mice displaying investigatory sniffing to almost all of the more concentrated odor dilutions tested. This is possibly counter-intuitive to what one may expect; when mice detect a novel odor they would display investigatory sniffing which throughout subsequent trials should habituate. There may be two reasons for this, each worthy of follow-up investigations to help better understand the influence of odor intensity on sniffing behaviors, which to-date has received considerably little attention (but see^46,50,51^,). One possibility is that as the intensity of a given odor increases, it is natural for ‘new’ odors to be perceived. For instance, a low concentration of tobacco odor may smell sweet but a high concentration would be pungent. In this view, animals may not habituate due to arousal with each new intensity being possibly perceived as a ‘new’ odor. The other simpler alternative is that averaging all data across mice (as necessary herein given the large dataset) may be masking overt habituation across individual animals who may have each detected the odor at a range of intensities. Nevertheless, our statistical outcomes indicate that synucleinopathy induced by PFFs seeding in the OB, is sufficient to perturb the normal detection of odors.

### Conclusion

This precise and rigorous methodology to assess odor sensitivity in PD animal models could be instrumental in determining how α-synuclein pathology affects features of olfactory perception and may help identify the neuropathological underpinnings of these deficits. Future work could assess olfactory deficits following OB injections with a serial dilution of PFFs to correlate olfactory impairment with α-synuclein pathology load, thereby allowing a more in-depth investigation into the relationship supported by the results in this study. Using more sensitive methodology also facilitates the development and evaluation of potential therapeutic interventions aimed to prevent olfactory deficits and the spreading of α-synuclein pathology, as well as the sequential neuronal dysfunction and loss.

## Supplementary information

Supplementary Information.
