# Supplementary material for: Deficits in olfactory sensitivity in a mouse model of Parkinson’s disease revealed by plethysmography of odor-evoked sniffing
Source: Sci Rep. 2020 Jun 8;10:9242. doi: 10.1038/s41598-020-66201-8 (PMC7280205; doi:10.1038/s41598-020-66201-8)
Supplement: Supplementary file 1 — Supplementary Information. [file 41598_2020_66201_MOESM1_ESM.pdf]

Deficits in olfactory sensitivity in a mouse model of Parkinson's disease revealed by plethysmography of odor-evoked sniffing

Michaela E. Johnson[1#], Liza Bergkvist[1#], Gabriela Mercado[1], Lucas Stetzik[1], Lindsay Meyerdirk[1], Emily Wolfrum[2], Zachary Madaj[2], Patrik Brundin[1*] and Daniel W. Wesson[3*]

Michaela E. Johnson: michaela.johnson@vai.org

Liza Bergkvist: liza.bergkvist@vai.org

Gabriela Mercado: gabriela.mercado@vai.org

Lucas Stetzik: lucas.stetzik@vai.org

Lindsay Meyerdirk: lindsay.meyerdirk@vai.org

Emily Wolfrum: emily.wolfrum@vai.org

Zachary Madaj: zachary.madaj@vai.org

Patrik Brundin: patrik.brundin@vai.org

Daniel W. Wesson: danielwesson@ufl.edu

[#] these authors contributed equally to this work

[*] shared corresponding authors

Author Affiliation:

[1-]Center for Neurodegenerative Science and [2-]Bioinformatics and Biostatistics Core, Van Andel Research Institute, Grand Rapids, MI 49503, [3-]Department of Pharmacology and Therapeutics, University of Florida, Gainesville, FL, USA

Corresponding Author:

Patrik Brundin

Email: patrik.brundin@vai.org

Daniel Wesson

Email: danielwesson@ufl.edu

Supplementary Table 1

Suppl. Table 1: Product details for the equipment required to create the olfactory testing apparatuses used herein.

item	vendor	cat#	quantity	URL
#10-32 Male Captivated O-Ring Plug, 5/16" Hex, ENP Brass. Pkg. of 10	Clippard	11782-7-ENP-PKG	1	https://www.clippard.com/part/11782-7-ENP-PKG
12V 1A power supply	anywhere		3	
2-way normally closed solenoid, 12V, 1/8" barb	Parker	003-0141-900	10	https://ph.parker.com/us/12051/en/series-3-miniature-inert-liquid-valve
3-way normally closed solenoid, 12V, 1/8" barb	Parker	003-0356-900	1	https://ph.parker.com/us/12051/en/series-3-miniature-inert-liquid-valve
40ml glass headspace vials (case of 100)	Shamrock Glass	6-01f	1+	https://www.shamrockglass.biz/640mlca28mm.html
5 Gal. Black Resin Thermoformed Nursery Pot	Home Depot	Store SKU #520359	2	https://www.homedepot.com/p/5-Gal-Black-Resin-Thermoformed-Nursery-Pot-TFR005G0G18/300434568
8 Pole Bessel Filter/Amplifier	Cygnus Technology Inc.	FLA-01	2	http://www.cygnustech.com/prices.html
Block Manifold #10-32 Threads, 1/16” ID Barbs, 12-Station	Clippard	BTT2-12	1	https://www.clippard.com/part/BTT2-12
Block Manifold #10-32 Threads, 1/8” ID Barbs, 12-Station	Clippard	BTT4-12	1	https://www.clippard.com/part/BTT4-12
BNC cable	anywhere		2	E.g. Belkin RG58 50-Ohm Thin Ethernet Coaxial Cable with BNC to BNC Male Connectors (6 Feet), https://www.amazon.com/Belkin-50-Ohm-Ethernet-Coaxial-Connectors/dp/B00004Z5KG/ref=sr_1_10?ie=UTF8&keywords=bnc%20cable&qid=1518814622&s=electronics&sr=1-10
Buxco® Small Animal Whole Body Plethysmograph	DSI	601-1425-001	2	https://www.datasci.com/products/buxco-respiratory-products/finepointe-whole-body-plethysmography

CED's Spike2 software	CED		1	http://ced.co.uk/products/spkovin
Cole-Parmer PVC Tubing, 1/8" x 1/4", 50 Ft/Pk	Cole-parmer	EW-96605-01	1	https://www.coleparmer.com/i/cole-parmer-pvc-tubing-1-8-x-1-4-50-ft-pk/9660501
Computer/laptop	anywhere		1	
CUI Desktop AC Adapter, 84W 12V 7A, 2.1x5.5, Level VI	Mouser Electronics	490-SDI90-12-U-P	1	https://www.mouser.com/ProductDetail/CUI-Inc/SDI90-12-U-P5/?qs=/ha2pyFadugghadUNUPSuPd176nfrcZNskshP68UsgH51UEjKQoAgQ==
Flow transducer w temp and humidity	DSI	601-2233-001	2	https://www.datasci.com/products/buxco-respiratory-products/finepointe-whole-body-plethysmography
Flowmeter, SS float, max. flow rate 0.633 SCFH (299 ml/min) air	Dwyer	va1046	1	http://www.dwyer-inst.com/Product/Flow/Flowmeters/VariableArea/SeriesVA#ordering
Hole cap for vials (case of 100)	Shamrock Glass	6-03d	1+	https://www.shamrockglass.biz/60hocaptofit4.html
John guest fitting Rigid Elbow, 1/4" Tube OD x 1/8" NPTF Male, 10pk	Amazon		1	https://www.amazon.com/John-Guest-Acetal-Copolymer-Fitting/dp/B007COMALY/ref=sr_1_1?ie=UTF8&keywords=john%20guest%20fitting%20Rigid%20Elbow,%201/4%22%20Tube%20OD%20x%201/8%22%20NPTF%20Male,%2010pk&qid=1515006824&sr=8-1
LAB RAT EPHYS SYSTEM	TDT		1	https://www.tdt.com/system/lab-rat-ephys-system/
Male Luer to 500 Series Barb, 1/16" (1.6 mm) ID Tubing	Nordson medical	MLRL004-1	1+	https://www.nordsonmedical.com/Shop/Fluid-Management/Products/MLRL004-1
Male Luer to 500 Series Barb, 1/8" (3.2 mm) ID Tubing	Nordson medical	MLRL013-6005	1+	https://www.nordsonmedical.com/Shop/Fluid-Management/Products/MLRL013-6005
Oversized 440C Stainless Steel Bar 1/8" Thick, 1/2" Wide,	McMaster-Carr	9575K179	1	https://www.mcmaster.com/9575k179
Polyurethane Ribbon Hose, 1/4" OD-1/8" ID, Multi-Color, 50' Roll	Clippard	URH8-0804-02T-050	1+	https://www.clippard.com/part/URH8-0804-02T-050

Polyurethane Ribbon Hose, 1/8" OD-1/16" ID, Multi-Color, 50' Roll	Clippard	URH8-0402-02T-050	1+	https://www.clippard.com/part/URH8-0402-02T-050
Push-Quick Male Compact Connector, 1/4", #10-32, Pack of 10	Clippard	PQ-CC08N-PKG	2	https://www.clippard.com/part/PQ-CC08N-PKG
Stereo to bnc adapter	anywhere		1	E.g. MyCableMart 4 INCH Right Angle 3.5mm Mono Male to Female BNC Adapter Cable, https://www.amazon.com/MyCableMart-RIGHT-FEMALE-Adapter-Cable/dp/B01N51QBRH/ref=sr_1_1?dpID=31e5-SWNQhL&dpSrc=srch&ie=UTF8&keywords=stereo%20to%20bnc%20adaptor&preST=_SX300_QL70_&qid=1518814043&sr=8-1
Teflon septa for headspace vials (case of 100)	Shamrock Glass	6-04d	1+	https://www.shamrockglass.biz/6setetofit15.html
Tetra 77851 whisper air pump 10 gallon	Amazon	77851	3	https://www.amazon.com/Tetra-77851-Whisper-Pump-10-Gallon/dp/B0009YJ4N6/ref=sr_1_1?ie=UTF8&keywords=77851%20tetra&qid=1515007053&s=industrial&sr=8-1
Valve driver board	LabJack	ps12dc	1	https://labjack.com/accessories/ps12dc-power-switching-board
Marpac Dohm Classic White Noise Sound Machine, White	Amazon		1	https://www.amazon.com/Marpac-Classic-White-Noise-Machine/dp/B00HD0ELFK/ref=sr_1_6_a_it?ie=UTF8&keywords=white%2Bnoise&qid=1539337504&sr=8-6&th=1

Supplementary Figure 1

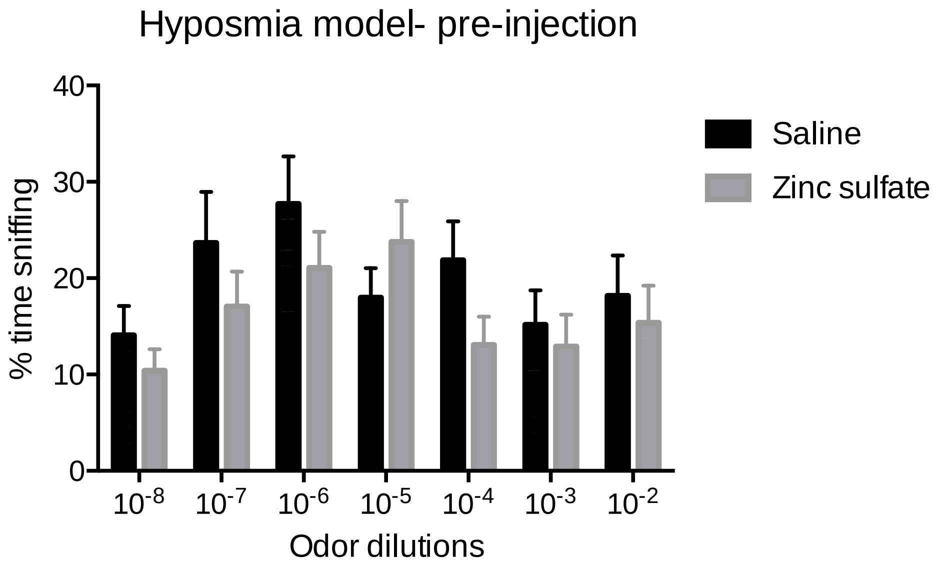

Suppl. Fig. 1. Validation of the semi-automated olfactometer. This graph represents pooled results in response to methyl valerate and 1,7-Octadiene odors. Male wild type mice allocated to saline or the zinc sulfate group spent a similar amount of time engaged in investigatory sniffing prior to intranasal injections. Data displayed as mean ± SEM, n = 9 per treatment group.

Supplementary Figure 2

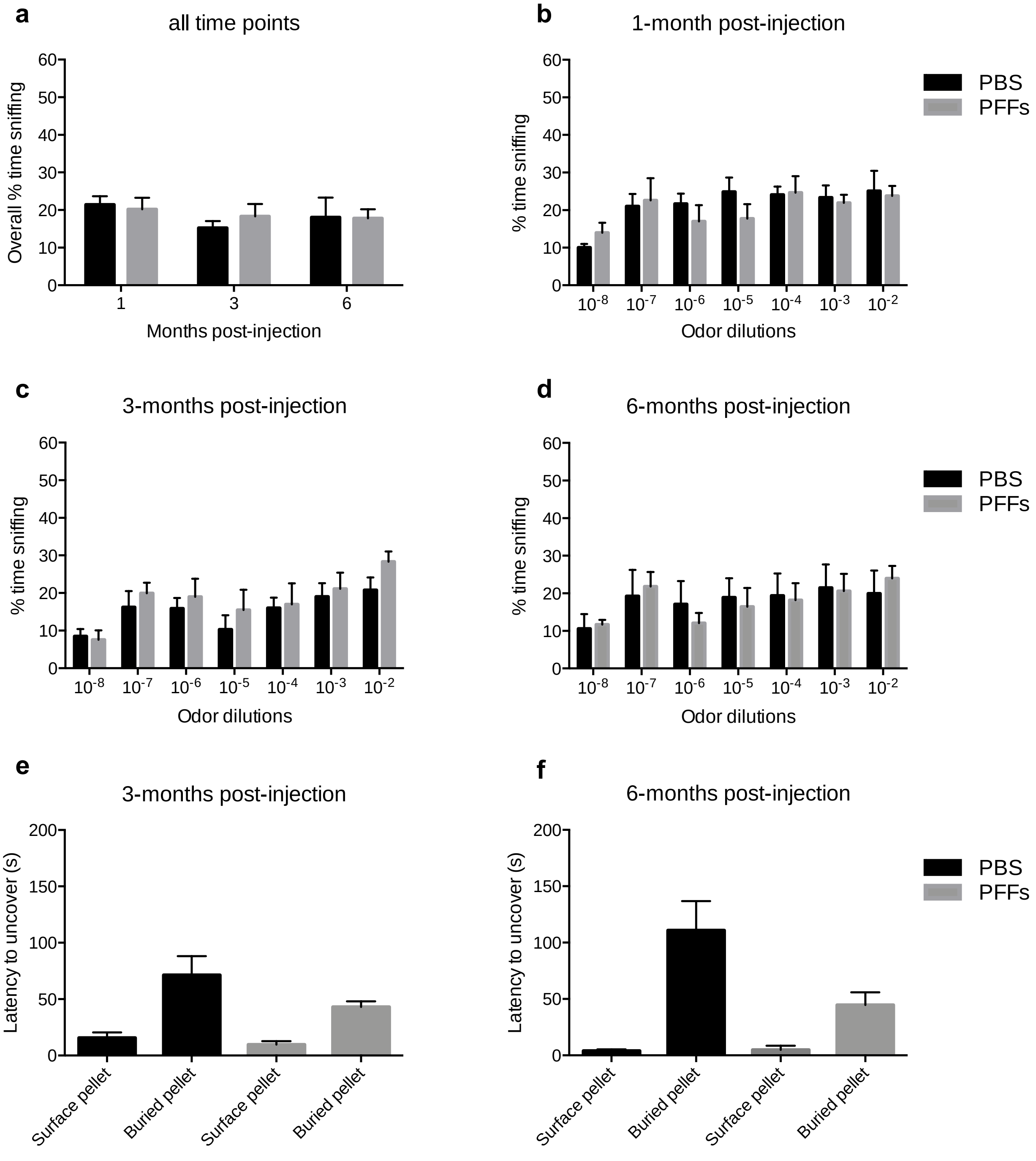

Suppl. Fig. 2. Olfactory deficits in male mice injected with PFFs. Graphs a-d represent pooled results in response to heptanal, isoamyl acetate and 1,7-Octadiene odors. Male wild type mice receiving bilateral PFFs OB injections spent a similar amount of time engaged in overall investigatory sniffing (a) compared to the PBS group at one- (b), three- (c) and six-months (d)

post-injection. No difference in olfaction was detected for mice receiving bilateral PFFs OB injections using the buried pellet test at three- (e) and six-months (f) post-injection. Data displayed as mean $\pm$ SEM, n = 5 for the PFF group and n = 6 for the PBS group.
